# Vav3 oncogene activates estrogen receptor and its overexpression may be involved in human breast cancer

**DOI:** 10.1186/1471-2407-8-158

**Published:** 2008-06-02

**Authors:** Kiwon Lee, Yin Liu, Jun Qin Mo, Jinsong Zhang, Zhongyun Dong, Shan Lu

**Affiliations:** 1 Department of Pathology, University of Cincinnati College of Medicine, 2120 E. Galbraith Road, Cincinnati, OH 45237, USA; 2 Department of Cancer and Cell Biology, University of Cincinnati College of Medicine, 231 Albert Sabin Way, Cincinnati, OH 45267, USA; 3 Department of Medicine, University of Cincinnati College of Medicine, 231 Albert Sabin Way, Cincinnati, OH 45267, USA

## Abstract

**Background:**

Our previous study revealed that Vav3 oncogene is overexpressed in human prostate cancer, activates androgen receptor, and stimulates growth in prostate cancer cells. The current study is to determine a potential role of Vav3 oncogene in human breast cancer and impact on estrogen receptor a (ERα)-mediated signaling axis.

**Methods:**

Immunohistochemistry analysis was performed in 43 breast cancer specimens and western blot analysis was used for human breast cancer cell lines to determine the expression level of Vav3 protein. The impact of Vav3 on breast cancer cell growth was determined by siRNA knockdown of Vav3 expression. The role of Vav3 in ERα activation was examined in luciferase reporter assays. Deletion mutation analysis of Vav3 protein was performed to localize the functional domain involved in ERα activation. Finally, the interaction of Vav3 and ERα was assessed by GST pull-down analysis.

**Results:**

We found that Vav3 was overexpressed in 81% of human breast cancer specimens, particularly in poorly differentiated lesions. Vav3 activated ERα partially via PI3K-Akt signaling and stimulated growth of breast cancer cells. Vav3 also potentiated EGF activity for cell growth and ERα activation in breast cancer cells. More interestingly, we found that Vav3 complexed with ERα. Consistent with its function for AR, the DH domain of Vav3 was essential for ERα activation.

**Conclusion:**

Vav3 oncogene is overexpressed in human breast cancer. Vav3 complexes with ERα and enhances ERα activity. These findings suggest that Vav3 overexpression may aberrantly enhance ERα-mediated signaling axis and play a role in breast cancer development and/or progression.

## Background

Vav3 oncogene, a quanine nucleotide exchange factor (GEF) for Rho family GTPases, belongs to Vav family proteins. The three mammalian Vav proteins (Vav1, Vav2, and Vav3) differ in their tissue distribution. Vav1 is primarily expressed in hematopoietic cells, while Vav2 and Vav3 are more ubiquitously expressed [1,2]. Vav proteins contain multiple function motifs and are involved in various cellular signaling processes, including cytoskeleton organization, calcium influx, phagocytosis, and cell transformation [3]. Vav proteins share a common structure, including a N-terminal calponin homology (CH) domain involved in Ca^+2 ^mobilization and transforming activity, an acidic domain (AD) containing three regulatory tyrosines, a Dbl homology (DH) domain with a conserved region that promotes the exchange of GDP for GTP on Rac/Rho GTPases, a pleckstrin homology (PH) domain binding to PIP_3 _that enables its movement to the inner face of the plasma membrane, two Src-homology 3 (SH3) domains interacting with proteins containing proline-rich sequences, and a Src-homology 2 (SH2) domain interacting with proteins containing phosphorylated tyrosines [4,5]. Tyrosine phosphorylation by receptor protein tyrosine kinase or cytoplasmic protein tyrosine kinase is required for Vav protein activation. In the non-phosphorylation state, Vav is folded, which is achieved by binding of the tyrosines in the AD domain to the DH domain and binding of the CH domain to the C1 region. Upon phosphorylation of the tyrosines in the AD domain, the folding is opened and the DH domain is exposed. Thus, Vav protein is activated and interacts with substrate proteins, and the PH domain is exposed for PIP3 binding [6].

Breast cancer is the most common malignant disease worldwide and the number one cause of cancer-related death among non-smoking women in the US. The major problem in breast cancer therapy is development of estrogen-insensitive growth after hormonal therapy. Aberrant ERα activation by various mechanisms contributes to breast cancer development and estrogen-resistant diseases [7-9]. This ERα hypersensitivity can be achieved by estrogen-independent mechanisms, such as ERα phosphorylation by crosstalking with signal transduction pathways and overexpression of nuclear receptor coactivator SRC3 [10,11]. Numerous studies have shown that EGFR/HER2-elicited signaling is involved in human breast cancer [9]. In addition, elevated PI3K-Akt signaling, mediated by PTEN deletion and/or mutation and PI3K subunit p110a (PI3KCA) mutation, upregulates ERα activity and is correlated with the breast cancer development and anti-estrogen resistance [12-15]. Activation of PI3K has been implicated in part because the downstream PI3K target, Akt, phosphorylates and promotes ligand-independent ERα activation [16,17]. Transgenic breast cancer mouse models have confirmed that elevated signaling in the EGFR/HER2-PI3K-Akt pathway either by targeted Akt overexpression or HER2 overexpression in breast epithelial cells induces breast cancer development [18-20]. These signaling pathways have been the targets for breast cancer therapy [7-9].

The classical ERα is a ligand-dependent transcription factor that activates transcription of its target genes in nucleus, which is known as genomic ERα activity. Recent findings revealed that the classical steroid hormone receptors also associate with cell membrane and mediate cell signaling through kinase cascade, defined as nongenomic activity [21,22]. Nongenomic ERα resides in multiprotein complexes with molecules, such as MNAR/PELP1 and src, in the cytoplasm and signals through the PI3K-Akt and MAPK pathways in breast cancer cells [23,24]. Nongenomic ERα signaling has been shown to contribute to estrogen-independent growth in breast cancer.

Recently, we and others found that Vav3 oncogene is overexpressed in androgen-independent prostate cancer cells, enhances androgen receptor (AR) activity, and stimulates androgen-independent growth in prostate cancer cells [25,26]. We further showed that Vav3, as a signal transducer, upregulates AR activity partially via PI3K-Akt signaling [25]. The DH domain of Vav3 is responsible for AR activation. Vav3 also potentiates EGF activity for cell growth and AR activation in prostate cancer cells. More importantly, Vav3 is overexpressed in 32% of human prostate cancer. These findings suggest that Vav3 overexpression may be involved in prostate cancer.

The purpose of this study is to determine the role of Vav3 in breast cancer. We found that Vav3 is overexpressed in human breast cancer specimens and cell lines. Vav3 stimulates growth of breast cancer cells and activates ERα partially via PI3K-Akt signaling. Vav3 potentiates EGF activity for cell growth and ERα activation in breast cancer cells. These data suggest that Vav3 impacts on ERα signaling axis and its overexpression may be involved in breast cancer.

## Methods

### Reagents

RPMI 1640 medium was purchased from Invitrogen (Gaithersburg, MD). Fetal bovine serum (FBS) and charcoal/dextran-treated FBS were purchased from HyClone Laboratories (Logan, UT). Human Vav3-specific Stealth™ Select RNAi (siVav3-247: 5'-CCCAGTTTCTCTGTTTGAAGAACAT-3') and its control (control-247: 5'-CCCTTCTCTGTTTGTAAAGAGACAT-3') were designed by a software in Invitrogen website and we purchased both oligos from Invitrogen as described before [25]. The transfection reagent Lipofectamine™ 2000 was from Invitrogen. Anti-Vav3 and anti-ERα antibodies were obtained from Upstate Biotechnology (Charlottesville, VA).

### Cell culture

The human breast cancer cell lines MCF7 and T47D, and cervical carcinoma cell line Hela were obtained from ATCC (Rockville, MD) and maintained in RPMI-1640 medium supplemented with 10% FBS (complete medium) at 37°C in 5% CO2. Nontumoral breast epithelial MCF-10A cells were obtained from ATCC. Transient transfection experiments were performed in RPMI-1640 medium supplemented with 10% charcoal/dextran-treated FBS (stripped medium).

### Plasmids

Plasmids pBEF-Vav3, pHEF-Vav3*, pHEF-Vav3*-ΔDH, pHEF-Vav3*-ΔSH, and control vector pHEF were detailed in our previous studies [25]. pS2-Luc is a gift from Dr. Sohaib Khan, Department of Cell Biology, University of Cincinnati College of Medicine. ERα and ERE-Luc are gifts from Dr. Zafar Nawaz, Braman Breast Cancer Institute, University of Miami Miller School of Medicine.

For generation of GST-Vav3-DH+PH construct, we designed upper primer 5' CGA*GAATTC*AAGGCAGAGGAAGCACATCAG containing Eco RI site and lower primer 5' TCT*GCGGCCGC*TGTTTAGGAGTTCTTCGCAG containing Not I site flanking both the DH and PH domains of Vav3 gene. The PCR product by amplification of the DH and PH domains using this pair of primers was subcloned into pGEX-4T-1 vector (GE Healthcare Bio-Sciences Corp. Piscataway, NJ) in frame by Eco RI and Not I sites.

### Cell growth assay

Tumor cell growth was estimated by MTT assay as previously described [27]. Briefly, breast cancer cells were seeded into 96-well cell culture plates at a density of 2.5 × 10^3 ^cells/well in stripped medium. After incubation in 5% CO2 at 37°C overnight, the cells were transfected with Vav3 siRNA and control siRNA using Lipofectamine 2000 and then cultured in stripped medium without or with E2 (10^-9^M) for 5 days. At the end of incubation, 20 ul of MTT (2.5 mg/ml in PBS) was added to each well, and the cells were further incubated for one hour at 37°C to allow complete reaction between the dye and the enzyme mitochondrial dehydrogenase in the viable cells. After removal of the residual dye and medium, 100 ul of dimethylsulfoxide was added to each well, and the absorbance at 570 nm was measured using BMG microplate Reader (BMG Labtech, Inc., Durham, NC).

### Western blot analysis

Western blot analysis was performed as previously described [27]. Briefly, aliquots of samples with the same amount of protein, determined using the Bradford assay (BioRad, Hercules, CA), were mixed with loading buffer (final concentrations of 62.5 mM Tris-HCl, pH 6.8, 2.3% SDS, 100 mM dithiothreitol, and 0.005% bromophenol blue), boiled, fractionated in a SDS-PAGE, and transferred onto a 0.45-um nitrocellulose membrane (BioRad). The filters were blocked with 2% fat-free milk in PBS, and probed with first antibody in PBS containing 0.1% Tween 20 (PBST) and 1% fat-free milk. The membranes were then washed four times in PBST and incubated with horseradish peroxidase-conjugated secondary antibody (BioRad) in PBST containing 1% fat-free milk. After washing four times in PBST, the membranes were visualized using the ECL Western blotting detection system (Amersham Co., Arlington Height, IL). For western blot analysis of Vav3 expression, the first antibody was incubated overnight at 4°C.

### Reporter assay

Cells (10^5^/well) were seeded in 12 well tissue culture plates. Next day, Optifect-mediated transfection was used for the transient transfection assay according to the protocol provided by Invitrogen/Life Technologies, Inc. The cells were then treated with hormone or drugs in stripped medium for 24 hours. Subsequently, the cell extracts were prepared and luciferase activity was assessed in a Berthold Detection System (Pforzheim, Germany) using a kit (Promega, Madison, WI) following the manufacture's instruction. For each assay, cell extract (20 ul) was used and the reaction was started by injection of 50 ul of luciferase substrate. Each reaction was measured for 10 seconds in the Luminometer. Luciferase activity was defined as light units/mg protein.

### GST pull down

GST-Vav3-DH+PH and control GST vectors were transformed into BL21 bacteria, respectively (Protein Express, Inc. Cincinnati, OH). The transformed bacteria were cultured in L-Broth with addition of 100 uM of IPTG to induce GST-fusion protein expression. Then, the bacteria were harvested and subjected to GST fusion protein purification by Sonication and using Glutathione Sepharose 4B (Amersham Bioscience).

For pull down reaction, 5~10 ug of GST or GST-Vav3-DH+PH was incubated with 1 mg of cell extracts from MCF7 cells in a binding buffer [20 mM of Tris.CL, PH. 7.9; 300 mM of KCL; 0.05% of NP-40; 0.2 mM of EDTA; 20% of Glycerol; 1 mM of Dithiothritol; 1 mM of phenylmethylsulfonyl fluoride (PMSF), 1× of protease inhibitor cocktail (Roche Diagnostics)] for overnight [28,29]. Then, the beads were washed for five times in a washing buffer (20 mM of Tris.CL, PH. 7.9; 300 mM of NaCL; 0.01% of NP-40; 0.2 mM of EDTA; 20% of Glycerol; 0.5 mM of Dithiothritol) and boiled in 1 × SDS loading buffer. The proteins in the supernatant were subjected for SDS-PAGE, which was visualized by Coomassie Blue staining. The samples were also subjected to western blot analysis for ERα.

### Immunohistochemistry (IHC) staining

IHC staining was performed as detailed in our previous studies [25]. Briefly, paraffin-embedded section of breast cancer tissue array (US Biomax, Rockville, MD) was deparaffinized in xylene, rehydrated in graded alcohol, and transferred to PBS. The slides were treated with a citric acid-based antigen-retrieval buffer (DAKO Co., Carpinteria, CA), followed by 3% H_2_O_2 _in methanol, incubated in blocking buffer (5% BSA and 5% horse serum in PBS) and then in the blocking buffer containing antibodies against human Vav3 (Upstate Biotechnology Inc.). After washing, the slide was incubated with a biotinylated secondary antibody (BioGenex Laboratories, San Ramon, CA), followed by washing and incubation with the streptavidin-conjugated peroxidase (BioGenex). A positive reaction was visualized by incubating the slides with stable diaminobenzidine and counterstaining with Gill's hematoxylin (BioGenex) and mounted with Universal Mount mounting medium (Fisher Scientific, Pittsburgh, PA). The intensity and extent of cytoplasm-positive labeling for Vav3 in tissue arrays were assessed semiquantitatively and scored as 0 (no staining), 1+ (weak and focal staining in <25% of tissue), 2+ (moderate intensity in 25–50% of tissue), and 3+ (moderate intensity in >50% of tissue), and 4+ (strong and diffused staining in >50% of tissue).

## Results

### Expression analysis of Vav3 in human breast cancer specimens

**Figure 1:**
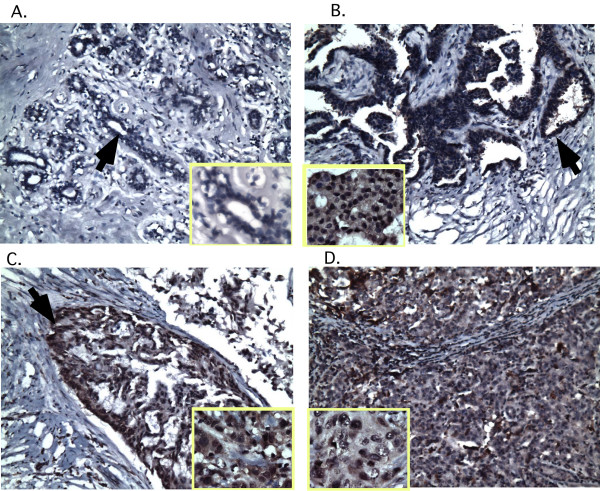
**Overexpression of Vav3 in human breast cancer.** (A) The normal breast epithelial cells reveal negative immunoreactivity for Vav3 (200× magnification). Breast adenocarcinomas with well-differentiation (B), moderately differentiation(C), and poorly differentiation (D) show positive immunoreactivity (brown staining) for Vav3 in both nucleus and cytoplasm. Arrows indicate the breast epithelial and cancer cells. The microphotographs indicate nucleus feature of the breast epithelial and breast cancer cells (400× magnification). For moderately and poorly differentiation breast cancer cells, the nuclei are significantly enlarged and hyperchromatic with coarse clumping of chromatin, prominent nucleoli, and irregular nuclear membrane.

To determine a potential role of Vav3 in breast cancer, we evaluated Vav3 expression in surgical specimens of human breast cancer by IHC analysis using anti-Vav3 antibody. No significant Vav3 immunoreactivity was detected in all normal breast tissue sections (0/8) (Table 1**and **Figure 1). In contrast, Vav3 staining, detected in both cytoplasm and nucleus of the epithelial cells but not in stroma of breast tissues, was found in 35 out of 43 tumor tissue sections (35/43, 81%, *p *< 0.0001). Among the tumor sections, Vav3 staining was found in approximately 67% (27/35) of the specimens with well to moderately differentiated tumors and 100% (8/8) of those with poorly differentiated tumors, respectively. The statistical analysis of association of Vav3 overexpression with poorly differentiated breast tumors failed (*p *> 0.05), possibly due to the small number of poorly differentiated tumor specimens. The proportion of sections with higher intensity of Vav3 staining, however, was significantly elevated in the specimens with poorly differentiated tumors (*p *< 0.01). As shown in Figure 1, for moderately and poorly differentiation breast cancer cells, the nuclei are significantly enlarged, hyperchromatic with coarse clumping of chromatin, prominent nucleoli, and irregular nuclear membrane.

**Table 1:** IHC analysis of Vav3 expression in breast cancer specimens.

Differentiation	Staining intensity			Intensity (>1)
	Negative	1–2	3–4	

Well to moderate	8	22	5	27/35
Poor	0	2	6	8/8
Normal breast tissue	8	0	0	0/8
Normal vs. tumor				p < 0.0001
Well to moderate vs poor differentiation				p > 0.05

Differences in the IHC staining of surgical specimens of human breast cancer were analyzed with the chi-square test.

**Figure 2:**
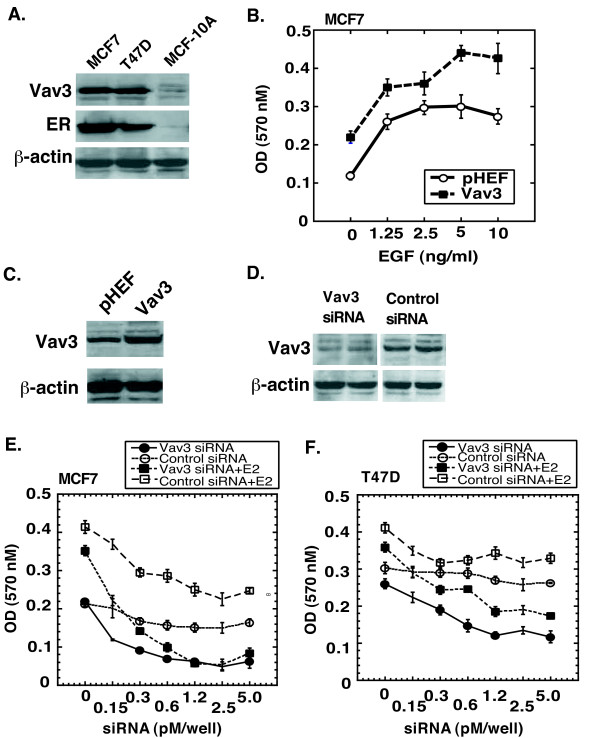
**Vav3 is involved in growth of breast cancer cells.** (A) Expression analysis of Vav3 and ERα in breast cancer MCF7 and T47D cells and nontumoral breast epithelial MCF-10A cells. The cell extracts were prepared from T47D, MCF7, and MCF-10A cells and subjected to western blot analysis for Vav3 and ERα. β-actin was served as loading control. (B) MCF7 cells were transiently transfected with Vav3 expression vector or control empty vector and then cultured in stripped medium in the absence or presence of EGF for 5 days, followed by MTT assay. The data was presented as absorbance at OD 570 nM. (C) MCF7 cells were transiently transfected with Vav3 expression vector or empty pHEF vector for 3 days, followed by cell extracts preparation and western blot analysis for Vav3. β-actin was served as loading control. (D) Knock down expression of Vav3 upon transfection of Vav3 siRNA. T47D cells were transfected with 5 pM/well of siVav3-247 or control-247 in 6-well plate for 3 days. The cell extracts were prepared and subjected to western blot analysis for Vav3. β-actin was served as loading control. (E and F) MCF7 cells and T47D cells (2500 cells/well in 96-well plate) were transiently transfected with siVav3-247 or control-247 at the concentrations of 0, 0.15, 0.3, 0.6, 1.2, 2.5, 5.0 pM/well using Lipofectamine 2000 for overnight. Then, the cells were cultured in stripped medium without or with E2 (10^-9 ^M) for 5 days, followed by MTT assay. The data was presented as absorbance at OD 570 nM.

Immunoblotting analysis also revealed that Vav3 expression was elevated in MCF-7 and T47D breast cancer cells in comparison with that in nontumoral breast epithelial MCF-10A cells (Figure 2A). ERα was detected in the two breast cancer cell lines, but not at the very low level in nontumoral MCF-10A cells.

### Vav3 is involved in growth of breast cancer cells

To determine a potential role of Vav3 in growth of breast cancer cells, we determined the role of Vav3 on estrogen-independent growth in breast cancer cells. Control and Vav3-transfected MCF7 cells were incubated in stripped medium in the presence of increasing concentrations of EGF. We found that Vav3 overexpression stimulated MCF7 cell growth in the absence of EGF and also significantly potentiated the cell growth in response to EGF treatment in a dose-dependent manner (Figure 2B. Elevated expression of Vav3 upon transient transfection of Vav3 expression vector was confirmed by western blot analysis in these cells (Figure 2C).

We then determined whether knockdown expression of Vav3 inhibits growth of these breast cancer cells using Vav3 siRNA that has been characterized previously [25]. Lipofectamine 2000 used for transfection of siRNA has been shown to knock down more than 80% activity of the endogenous gene in a panel of cells tested (Invitrogen). We confirmed a reduced level of Vav3 upon transfection of Vav3 siRNA by protein expression analysis relative to the control (Figure 2D). A growth stimulatory effect in response to estrogen stimulation was observed in T47D and MCF7 cells (Figure 2E**and **2F). We found that knockdown expression of Vav3 significantly inhibited both estrogen-dependent and -independent growth in these breast cancer cells (Figure 2E**and **2F). At 1.2 pM of siRNA, the growth of T47D and MCF7 cells was inhibited by 64% and 68% in the absence of estrogen and 46% and 77% in the presence of estrogen relative to their respective controls. These data suggest that Vav3 is involved in both estrogen-dependent and -independent growth in breast cancer cells.

### Vav3 upregulates ERα activity

Previous studies have revealed that Vav3*, a Vav3 mutant with N-terminal domain deletion including the AD domain containing three tyrosine residues, is a constitutive active form and has much stronger oncogenic effect relative to that by wild type Vav3 [30]. Structure analysis of Vav protein suggested that in the non-phosphorylation state, Vav protein is folded, which is achieved by binding of tyrosines in the AD domain to the DH domain and binding of the CH domain to the C1 region [6]. Phosphorylation of the tyrosines in the AD domain results in unfolding of Vav protein and exposure of the DH domain for interacting with substrate proteins. Consistently, we found that the constitutive active Vav3* shows a much stronger activity in upregulation of AR activity than that by Vav3 [25].

**Figure 3:**
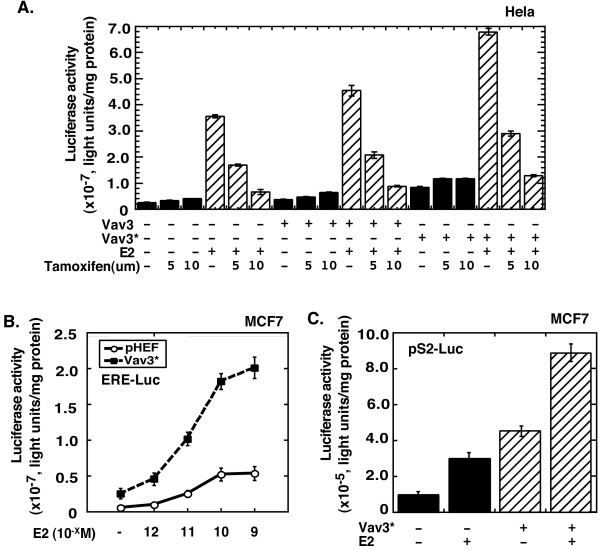
**Vav3 enhances ERα activity.** (A) Hela cells (10^5 ^cells/well in 12-well plate) were cotransfected with ERE-Luc (0.5 ug), expression vectors for Vav3, Vav3*, or empty vector pHEF (200 ng) and ERα (50 ng), respectively. Then, the cells were treated without or with E2 (10^-9 ^M) and without or with Tamoxifen. (B and C) MCF7 cells (10^5 ^cells/well in 12-well plate) were cotransfected with ERE-Luc (0.5 ug) (B) or pS2-Luc (0.5 ug) (C), and expression vector (0.25 ug) for Vav3*, or empty vector pHEF, respectively. Then, the cells were treated with E2. All transfection and drug treatment are in stripped medium for 24 hours, followed by luciferase assay. Renilla luciferase as an internal control was used to normalize the data. Data are presented as the mean (± SD) of duplicate values of a representative experiment that was independently repeated for five times.

To determine whether Vav3 regulates ERα activity, we used both Vav3 and Vav3* for the reporter assay. Hela cells were transiently cotransfected with a luciferase reporter driven by the estrogen response element (ERE), ERα expression vector, and either Vav3 or Vav3* expression vector. Overexpression of Vav3, and Vav3* with a greater effect relative to the empty vector pHEF, enhanced both basal and estrogen-stimulated ERα activity (Figure 3A). Furthermore, we found that both estrogen-stimulated and Vav3-activated ERα activities were blocked by ER antagonist Tamoxifen (Figure 3A). The inhibitory effect for ERα by Tamoxifen was compromised with Vav3 overexpression. Upregulation of the endogenous ERα activity by Vav3 relative to the empty vector pHEF was also confirmed in MCF7 cells by stimulation of luciferase reporter expression driven by ERE (Figure 3B) and natural promoter of ERα target gene pS2 (Figure 3C). Vav3 also significantly enhanced ERα activity stimulated by sub-physiological concentrations of estrogen (10^-11 ^and 10^-10 ^M) (Figure 3B). These data suggest that Vav3 overexpression confers ERα hypersensitivity.

**Figure 4:**
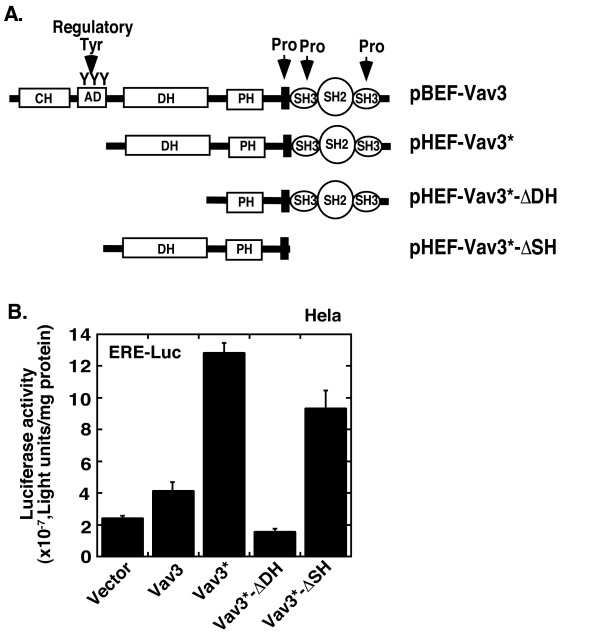
**Determination of the functional domain of Vav3 involved in ERα activation.** (A) Deletion constructs of Vav3. (B) Hela cells (10^5 ^cells/well in 12-well plate) were cotransfected with ERE-Luc (0.5 ug) and expression vectors (200 ng) for Vav3, Vav3*, Vav3*-ΔDH, Vav3*-ΔSH, or empty vector pHEF, as well as expression vector (50 ng) for ERα, respectively. All transfection and drug treatment are in stripped medium for 24 hours, followed by luciferase assay. Renilla luciferase as an internal control was used to normalize the data. Data are presented as the mean (± SD) of duplicate values of a representative experiment that was independently repeated for five times.

**Figure 5:**
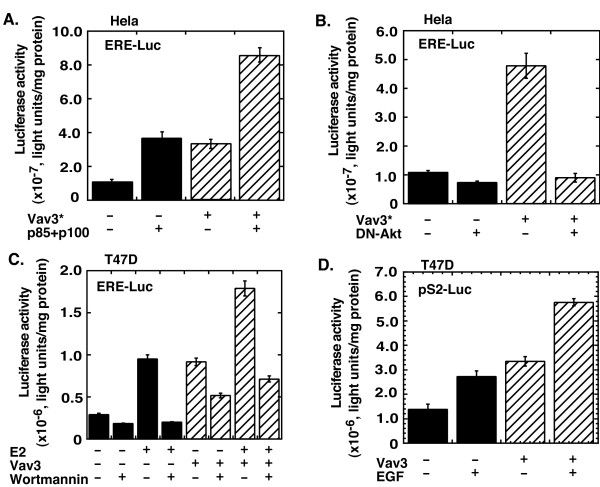
**Vav3 activates ERα partially via PI3K-Akt signaling and potentiates EGF for ERα activation.** (A) Hela cells were cotransfected with ERE-Luc (0.25 ug), expression vectors ERα (25 ng), Vav3* or empty vector pHEF (50 ng), p85+p110 or empty vector pCR3.1 (50 ng). (B) Hela cells were cotransfected with ERE-Luc reporter (0.25 ug/well in 12-well plate), expression vectors ERα (25 ng), dominant-negative Akt expression vector or empty vector pCR3.1 (0.1 ug), and Vav3* expression vector or empty vector pHEF (0.1 ug), respectively. (C) T47D cells were cotransfected with ERE-Luc (0.5 ug) and expression vector for Vav3 or empty vector pHEF (0.25 ug). Then, the cells were treated with Wortmannin (0.5 um). (D) T47D cells were cotransfected with pS2-Luc (0.5 ug) and expression vector for Vav3 or empty vector pHEF (0.25 ug). Then, the cells were treated with EGF (20 ng/ml). All transfection and drug treatment are in stripped medium for 24 hours, followed by luciferase assay. Renilla luciferase as an internal control was used to normalize the data. Data are presented as the mean (± SD) of duplicate values of a representative experiment that was independently repeated for five times.

We next examined the domains of Vav3 required for upregulation of ERα activity by analyzing both wild type Vav3 and Vav3 deletion mutants (Figure 4A). We found that Vav3* demonstrated a much stronger activity for ERα activation relative to Vav3 as compared with the empty vector pHEF, supporting the previous observation that activity of Vav3 is subjected to regulation by phosphorylation (Figure 4B**and **Figure 5D) [6]. In addition, deletion of the DH domain, but not the SH domain or the CH domain, abolished Vav3 function for ERα activation (Figure 4B). The truncated Vav3 protein with deletion of both PH and SH domains failed to activate ERα. Therefore, consistent with our previous observation of its effect for AR, the DH domain of Vav3 is also essential for ERα activation [25].

### Vav3 enhances ERα activity partially via the PI3K-Akt pathway and potentiates EGF for ERα activation

Elevated PI3K-Akt signaling enhances ERα activity and is thought to be critical for breast cancer development and progression [11,31,32]. Since Vav3 is a potential upstream regulator of the PI3K-Akt pathway [33,34], we investigated whether Vav3 overexpression impacts on PI3K-Akt signaling-mediated ERα activation. Hela cells were transiently cotransfected with ERE-Luc reporter, and expression vectors ERα, Vav3*, and p85 (the regulatory subunit of PI3K) and p110 (the catalytic subunit of PI3K). As shown in Figure 5A, cotransfection of Vav3* or PI3K relative to the empty vector pHEF or pCR3.1 stimulated ERα activity. ERα activity was further enhanced in cells cotransfected with both Vav3* and PI3K. On the other hand, cotransfection of a dominant-negative Akt relative to the empty vector pCR3.1 blocked Vav3*-mediated ERα activation (Figure 5B). Next, we determined the role of endogenous PI3K in Vav3-induced ERα activation. Data in Figure 5C showed that PI3K inhibitor Wortmannin blocked both basal and Vav3-stimulated ERα activation in the absence and presence of E2. Similar results were also noted in cells treated with PI3K inhibitor LY294002 (data not shown). These data indicate that Vav3 activates ERα at least partially via PI3K-Akt signaling.

In agreement with its effects on estrogen-independent growth in breast cancer cells (Figure 2B), overexpression of Vav3 potentiated EGF-stimulated ERα activation (Figure 5D). This data suggests that Vav3 overexpression may mediate EGFR/HER2/neu-elicited signaling leading to ERα hypersensitivity.

### Vav3 complexes with ERα and impacts on ERα signaling axis

**Figure 6:**
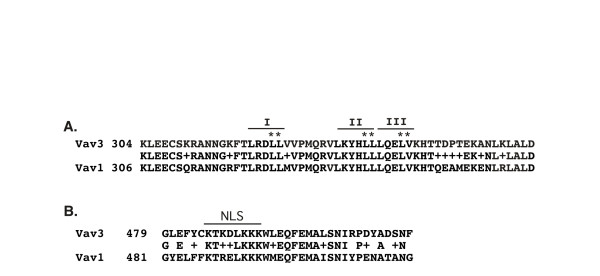
**Sequence analysis of Vav3 and Vav1 genes.** (A) The consensus sequences of LXXLL motifs I, II, and III were identified in the DH domain of Vav3 as indicated. Mutation of LLLQELV sequence overlapping with LXXLL motifs II and III has been shown to abolish the GEF activity of Vav3. (B) A consensus sequence of the nucleus localization signal (NLS) in Vav3 was localized in the PH domain.

Recent findings implicate that Vav family proteins also complex with transcription factors and regulate gene expression [35,36]. A nuclear localization signal (NLS) in the PH domain was shown to be solely responsible for the nucleus localization of Vav1 protein, indicating a role of Vav family protein as a transcription coregulator. We have demonstrated that the DH domain of Vav3 is essential for AR and ERα activation (Figure 4) [25]. We performed sequence analysis of Vav proteins and found that the DH domain of Vav3 contains three consensus sequences of the LXXLL motifs or NR boxes, which have been well characterized and involved in interaction with nuclear receptors (Figure 6A). In addition, homologous analysis of Vav3 and Vav1 genes identified a conserved NLS in the PH domain of Vav3 (Figure 6B). These findings suggest that Vav3 is also localized in nucleus and regulates transcription of nuclear receptors.

**Figure 7:**
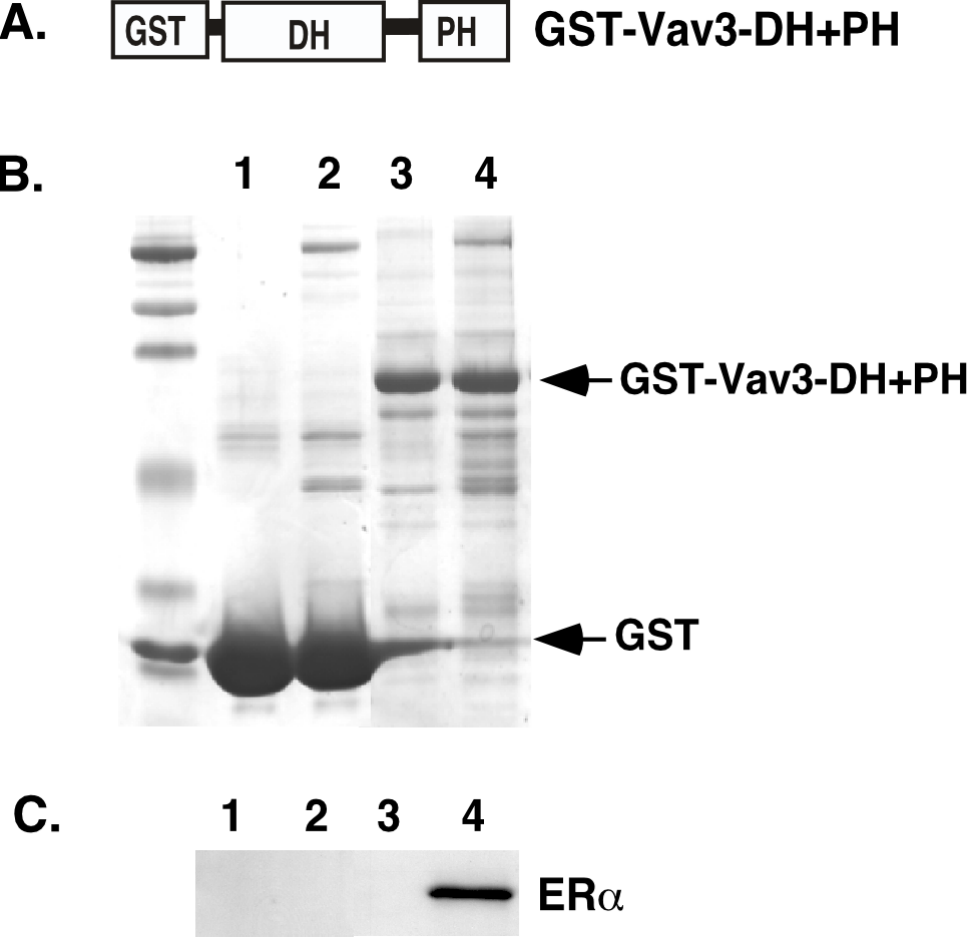
**Vav3 complexes with ERα by GST pull down analysis.** (A) GST-Vav3-DH+PH fusion protein. (B) GST-Vav3-DH+PH fusion protein (lane 3 and 4) and control GST protein (lane 1 and 2) were subjected to pull down reaction in the absence (lane 1 and 3) and presence (land 2 and 4) of cell extract derived from MCF7 cells. The samples were fractionated in SDS-PAGE and stained with Coomassie Blue. (C) The same samples were subjected to western blot analysis for ERα.

We then determined whether Vav3 complexes with ERα. We performed GST pull down experiment to confirm the interaction between Vav3 and ERα. A GST fusion protein including the DH and PH domain of Vav3 (GST-Vav3-DH+PH) was generated (Figure 7A). Cell extract derived from MCF7 cells was incubated with immobilized GST-Vav3-DH+PH fusion protein or GST protein. Then, the pull down samples were fractionated by SDS-PAGE (Figure 7B) and subjected to western blot analysis for ERα. We found that GST-Vav3-DH+PH fusion protein, but GST protein, interacted with ERα (Figure 7C). In summary, we found that Vav3 contains NLS in the PH domain and three LXXLL motifs in the DH domain. The deletion mutation and functional analysis by luciferase reporter assay showed that the DH domain of Vav3 is essential for enhancing ERα activity and is involved in complex with ERα. Our data suggest that Vav3 complexes with ERα and its overexpression enhances ERα signaling axis in breast cancer cells.

## Discussion

Previous studies from our group have demonstrated that Vav3 is overexpressed in human prostate cancer and potentiates AR signaling [25]. Breast cancer and prostate cancer are steroid-dependent tumors and share a significant similarity in their characteristics and treatment. For instance, growth of these cancer cells, mediated by their corresponding hormone receptors ERα and AR, is hormone-dependent. Hormone ablation is common therapy for both cancers. Recurrent diseases develop hormone-independent growth. Given that steroidal nuclear receptors share many common properties, we hypothesized that Vav3 may regulate ERα activity and is involved in human breast cancer. We tested this hypothesis in the present study by examining the expression of Vav3 in human breast cancer specimens and cell lines and investigated a potential role of Vav3 in breast cancer cell growth and ERα signaling. We found that Vav3 was overexpressed in human breast cancer, particularly in the poorly differentiated lesions and in the two most commonly used breast cancer cell lines. The knockdown expression of Vav3 compromised both estrogen-stimulated and -independent growth of breast cancer cells. On the other hand, overexpression of Vav3 enhanced ERα signaling. These data strongly suggest that Vav3 may play an important role in breast cancer development and/or progression.

Vav3 is an oncogene identified in cell transformation experiments [3]. Vav3 is activated upon ligand stimulation of EGF, insulin, Ros, and IGF receptors and physically associates with a variety of signaling molecules, including Rac1, Cdc42, PI3K, Grb2, and PLC-γ, leading to alteration in cell morphology and cell transformation [33]. Overexpression of Vav3 leads to PI3K activation and focus formation in NIH3T3 cells [34]. In contrast, blocking PI3K activation by PTEN and LY294002 inhibits Vav3-induced cell transformation. Furthermore, it has been shown that Vav3*, a Vav3 mutant with N-terminal domain deletion including the acidic domain, is a constitutive active form and has much stronger oncogenic effect compared with that by Vav3 [30]. Consistently, we found that the augmentation of ERα signaling by Vav3 overexpression was similarly inhibited by Wortmannin and by overexpression of a dominant-negative Akt. In addition, Vav3 showed a lower activity for ERα activation relative to that by Vav3*. EGF treatment significantly potentiated Vav3 activity for ERα activation. These data suggest that Vav3 is subjected to regulation by phosphorylation, most likely at the three tyrosines in the AD domain, which may cause conformation change, release the inhibitory effect of the N-terminal domain, and expose the DH domain for ERα binding.

Many coactivators of steroid hormone receptors have the LXXLL motifs, where L is leucine and X is any amino acid [37,38]. These coregulators interact with and upregulate ERα-mediated signaling in both nucleus and cytoplasm. For instance, SRC-1 and its related proteins are a family of coactivators containing the homologous bHLH-PAS domain and receptor-interacting domain (RID) with multiple LXXLL motifs and enhance transcription activity of nuclear receptors [37,38]. PELP1/MNAR containing the LXXLL motif interacts with and enhances both genomic and nongenomic ERα activities [21,24]. Recent findings implicate that Vav family proteins also complex with transcription factors and regulate gene expression. Vav1 was identified in the component of transcriptionally active nuclear factor of activated T cells (NFAT)- and nuclear factor NFkB-like complexes [35,36]. A nuclear localization signal (NLS) in the PH domain is solely responsible for nucleus localization of Vav1 protein, indicating a role of Vav family proteins as a transcription coregulator. We found that Vav3 contains the LXXLL motifs in the DH domain and NLS in the PH domain. This finding suggests that Vav3 is also a nuclear protein.

Previous study has shown that Vav protein can be activated by receptor tyrosine kinase upon activation of EGFR [39]. Furthermore, ERα resides in multi-protein complexes with molecules, such as MNAR/PELP1 and src, in the cytoplasm and signals through the PI3K-Akt and MAPK pathways in breast cancer cells [23,24]. ERα was also found localized in lipid rafts and involved in signaling elicited by EGFR and HER2 receptors [40,41]. We found that Vav3 activates ERα partially via PI3K-Akt signaling and potentiates EGF effect for cell growth and ERα activation in breast cancer cells. More interestingly, we found that Vav3 complexes with ERα. These findings suggest that Vav3 enhances ERα signaling axis in breast cancer cells. Vav3 overexpression may confer ERα hypersensitivity and play a role in breast cancer. Given both nuclear and cytoplasmic localization of Vav3 protein, our data implicate that Vav3 may impact on both genomic and nongenomic ERα activity. Furthermore, the relationship of Vav3 and ERα in the context of EGFR/HER2 and PI3K-Akt signaling is remained to be determined.

Our findings support the notion that Vav3 overexpression may play a role in breast cancer, based on the following reasons: 1) Vav3 is overexpressed and correlated with poorly differentiated tumors in human breast cancer; 2) Vav3 contains the LXXLL motifs and complexes with ERα; 3) Vav3 enhances ERα activity partially via the PI3K-Akt pathway; 4) Vav3 is a protein with multiple domains and functions, including the SH2 domain interacting with receptor protein tyrosine kinase, the PH domain binding PIP3 involved in association with the cell membrane, and the DH domain involved in interaction with ERα; 5) Vav3 potentiates EGF for cell growth and ERα activation. Taken together, these findings suggest that Vav3 overexpression enhances ERα-mediated signaling axis and may be involved in breast cancer.

Data presented in this report clearly show that Vav3 is overexpressed in human breast cancer and is involved in growth of breast cancer cells and ERα signaling. Whereas our data showed that Vav3 complexes with ERα, molecular mechanisms underlying the enhancement of ERα signaling remain to be elucidated. Nevertheless, data presented here strongly suggest a novel mechanism that potentially leads to ERα hypersensitivity and breast cancer development and/or progression.

## Abbreviations

ERα: estrogen receptor α; EGFR: epidermal growth factor receptor; HER2: human epidermal growth factor receptor; GEF: quanine nucleotide exchange factor; IHC: Immunohistochemistry; NLS: nuclear localization signal.

## Competing interests

The authors declare that they have no competing interests.

## Authors' contributions

KL generated GST fusion protein for pull down analysis. YL performed cell cultures, proliferation assays, Western blot analysis, and reporter assay. ZD performed IHC. JQM did histopathological analysis of breast cancer tissues. JZ provided plasmid and expertise in protein/protein interaction. SL contributed to conception and design of study and interpretation of data. All authors read and approved the final manuscript.

## Pre-publication history

The pre-publication history for this paper can be accessed here:


